# Tacholess Envelope Order Analysis and Its Application to Fault Detection of Rolling Element Bearings with Varying Speeds

**DOI:** 10.3390/s130810856

**Published:** 2013-08-16

**Authors:** Ming Zhao, Jing Lin, Xiaoqiang Xu, Yaguo Lei

**Affiliations:** 1 School of Mechanical Engineering, Xi'an Jiaotong University, Xi'an 710049, China; E-Mails: zhaomingxjtu@gmail.com (M.Z.); xuxiaoqiang@stu.xjtu.edu.cn (X.X.); 2 State Key Laboratory for Manufacturing Systems Engineering, Xi'an Jiaotong University, Xi'an 710049, China; E-Mail: yaguolei@mail.xjtu.edu.cn

**Keywords:** bearings fault diagnosis, generalized demodulation, tacholess order tracking, envelope order spectrum, adaptive short-time Fourier transform

## Abstract

Vibration analysis is an effective tool for the condition monitoring and fault diagnosis of rolling element bearings. Conventional diagnostic methods are based on the stationary assumption, thus they are not applicable to the diagnosis of bearings working under varying speed. This constraint limits the bearing diagnosis to the industrial application significantly. In order to extend the conventional diagnostic methods to speed variation cases, a tacholess envelope order analysis technique is proposed in this paper. In the proposed technique, a tacholess order tracking (TLOT) method is first introduced to extract the tachometer information from the vibration signal itself. On this basis, an envelope order spectrum (EOS) is utilized to recover the bearing characteristic frequencies in the order domain. By combining the advantages of TLOT and EOS, the proposed technique is capable of detecting bearing faults under varying speeds, even without the use of a tachometer. The effectiveness of the proposed method is demonstrated by both simulated signals and real vibration signals collected from locomotive roller bearings with faults on inner race, outer race and rollers, respectively. Analyzed results show that the proposed method could identify different bearing faults effectively and accurately under speed varying conditions.

## 1. Introduction

Rolling element bearings are critical mechanical components in rotating machinery and their failure may lead to fatal breakdown and significant economic losses. Hence, the fault detection of rolling element bearing has attracted considerable attention in recent years. Vibration signals collected from bearings carry rich information about their health condition. Therefore, the vibration-based diagnostic methods have received intensive study during the past decade [1,2].

When a fault occurs in a bearing, periodic or quasi-periodic impulses will appear in the waveform of the vibration signal, while the corresponding bearing characteristic frequencies (BCFs) and their harmonics emerge in the frequency domain [3]. For this reason, the detection of faults in rolling element bearings is traditionally achieved by identification of the BCFs from the measured vibration signal. However, the fault signal of bearing is often corrupted by the measurement noise and interferences coming from other machine components, such as gears, which makes the BCFs not easy to be recognized. To effectively diagnose faults occurring in bearings, a variety of signal processing techniques have been developed. For example, the envelope analysis method focuses on the low-amplitude high-frequency broadband signals characterizing bearing conditions and may minimize the effects of interfering signals within the selected frequency band [1,4]. The major challenge in the application of envelope analysis is how to choose the optimal band for the demodulation [5]. To address this issue, spectral kurtosis was intensively investigated by Antoni [6–8]. It is shown that spectral kurtosis can indicate not only transient components in the signal but also their locations in the frequency domain, and therefore provides a guideline for the optimal demodulation bandwidth selection in the envelope analysis. Other signal processing tools, such as wavelet analysis [9,10], empirical mode decomposition (EMD) [11–13], minimum entropy deconvolution (MED) [14,15] and stochastic resonance [16–18] have also been applied to the bearing fault detection in recent years.

Although the above mentioned methods successfully detect fault for bearings to some extents, most of those methods are based on the assumption of constant running speed. However, in practice, almost all of the bearings experience different kinds of speed variations. For instance, the rotating speed (RS) of vehicle bearing is varying with its running speed, the RS of wind turbine supporting bearing is fluctuating with the wind speed [19], and the RS of mining excavator bearing is affected by the external load. Under variable speed conditions, the repetition frequencies of impulses also vary with time and hence the corresponding envelope signals are non-stationary in nature. The direct application of frequency-based methods (such as envelope spectrum analysis, spectral correlation) to those vibration signals of bearings will lead to spectral smearing and false diagnosis [1,20].

Several methods have been developed to process non-stationary vibration signals in recent years [21–27]. Among those techniques, order tracking [1,20,28] is an effective approach which has been applied to the diagnosis of rolling element bearings successfully. Different from traditional frequency-based methods, order tracking resamples the vibration signal at constant angular increments of a reference shaft. In this way, the frequency modulation and spectral smearing due to speed variation will not be introduced in the angular domain. Meanwhile, this technique can be combined with synchronous averaging to remove the background noise and non-concerned signal components generated by the gear meshing. For this reason, order tracking is an effective tool for the fault detection of rolling element bearings under the varying speed conditions. However, it should be stressed that order tracking method requires a tachometer or an encoder to provide a phase reference signal, which not only increases the measurement cost, but also brings inconvenience in the installation and adjustment. In some cases, it is even impossible to install those sensors.

To overcome the shortcomings of conventional methods, a tacholess envelope order analysis technique is established in this paper. In this technique, a tacholess order tracking method is first proposed based on generalized demodulation transform. By using this method, the tacho information of the bearing could be recovered from the vibration signal itself. On this basis, envelope order spectrum is utilized to transform the non-stationary envelope signal in the time-domain into a cyclo-stationary signal in the angular domain. In this way, the smearing problem caused by speed variation is solved effectively. The rest of this paper is organized as follows. The principle and implementation of order tracking and generalized demodulation is briefly reviewed in Section 2. The procedure of proposed technique is given in Section 3. The effectiveness of the proposed method is validated by some simulations and experiments in Section 4 and Section 5, respectively. Finally, conclusions are drawn in Section 6.

## 2. Review of Order Tracking and Generalized Demodulation

As discussed above, order tracking involves resampling the vibration signal at constant increments of shaft angle. Hence, instantaneous phase of shaft, *φ*(*t*), must be known as a prior. Conventionally, *φ*(*t*) is estimated by utilizing an encoder or a tachometer mounted the shaft of interest. However, this hardware-based order tracking scheme is only suitable for the case when those sensors are available. With the advancement of signal processing, the extraction of *φ*(*t*) from vibration signal itself has attracted much attention [29,30].

Generally, the vibration signals from rolling element bearings contain not only the structure resonance frequency excited by impacts, but also the fundamental frequency and its harmonics of bearing shaft. Since all the rotating harmonics are phase-locked with respect to the rotation angle of the bearing shaft, naturally, the *φ*(*t*) could be estimated by calculating the instantaneous phase of one certain harmonic, say the *k* th, as follows:

(1) $$\varphi (t)=\frac{\varphi_{k}(t)}{k}$$

where *φ_k_*(*t*) denotes the instantaneous phase of the *k* th rotating harmonic.

**Figure 1.**
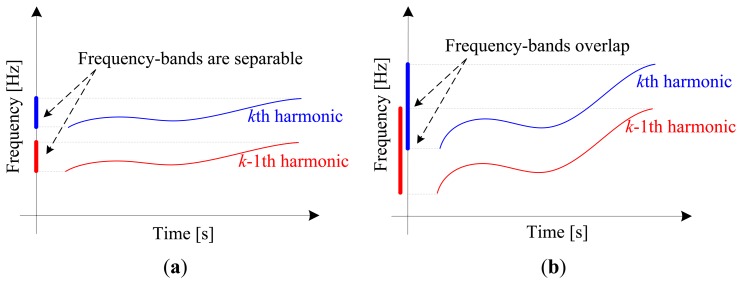
Time-frequency distribution and frequency-bands of harmonics: (**a**) Small speed variation; (**b**) Large speed variation.

However, the concept of instantaneous phase has physical meaning only for mono-component signal [31], which implies that the *k* th harmonic, *x_k_*(*t*), should be extracted from the raw vibration signal before calculating its instantaneous phase. In previous works [29,30], that harmonic is extracted by band-pass filtering. Unfortunately, this method is only applicable to small speed variation cases, where the frequency-bands of interested harmonic (the *k* th) and its adjacent harmonic (the *k*-1th) are separable in frequency domain, which is illustrated in Figure 1a. When the speed variation is large, the frequency-bands will overlap in the spectrum as illustrated in Figure 1b. Thus, it cannot be extracted by band-pass filtering. This deficiency significantly limits the application of order tracking to bearing fault diagnosis. In order to address this drawback, generalized demodulation is introduced to in this work.

Generalized demodulation is a novel signal processing tool, which is especially suitable for the separating of non-stationary multi-component signals [32–34]. The key of the generalized demodulation lies in the development of a signal transform, *i.e.*, generalized Fourier transform, which could transform the curved instantaneous frequency trajectory of an interested component into a line parallel to the time axis, thus avoiding overlap with other components in the frequency domain. This property makes it possible to separate any interested component from the non-stationary raw signal.

For a signal *x*(*t*), the generalized Fourier transform (GFT) is defined as [32]:

(2) $$X_{G}(f)=\int_{-\infty }^{\infty }x(t)e^{-j2\pi [ft+s_{0}(t\text{)}]}dt$$

where e^-^*^j^*^2π^^*s*_0_^^(^*^t^*^)^ is the transform kernel, and *s*_0_(*t*) is a real-valued phase function depending on the time only. It can be seen from Equation (2) that GFT is the same as the traditional Fourier transform when *x*(t)e^-^*^j^*^2π^^*s*_0_^^(^*^t^*^)^ is considered as the analyzed signal. When *s*_0_(*t*) = 0, GFT degrades into Fourier transform. From this point of view, the Fourier transform can be considered as a special case of GFT. Like the Fourier transform, the inverse GFT is defined as:

(3) $$x(t)=\int_{-\infty }^{\infty }X_{G}(f)e^{j2\pi [ft+s_{0}(t)]}df=e^{j2\pi s_{0}(t)}\int_{-\infty }^{\infty }X_{G}(f)e^{j2\pi ft}df$$

If we assume *X_G_*(*f*)= *δ*(*f*−*f*_0_), then *x*(*t*)= e^-^*^j^*^2π[^^*f*_0_^^+^^*s*_0_^^(^*^t^*^)]^ It implies that a signal with curved IF, *i.e.*, $f(t)=f_{0}+s_{0}^{\prime }(t)$, will be mapped to a linear IF path that parallel to the time axis by applying appropriate generalized Fourier transform. More specifically, if we wish to transform a signal with curved IF, *f*(*t*), into constant frequency of *f*_0_, we simply need to specify a function *s*_0_(*t*), which satisfies:

(4) $${s_{0}}^{'}(t)=f(t)-f_{0}$$

where $s_{0}^{'}(t)=ds_{0}(t)/dt$.

According to this property of generalized demodulation, the *k*th harmonic, *x_k_*(*t*), could be extracted from the raw vibration signal *x*(*t*) by the following procedures:

1. Although the definition of GFT is applicable to both real signal and analytic signal, however, the latter one is preferred in practice. It is due to the fact that the interference on the time-frequency plane caused by meaningless negative frequency could be avoided when the signal is analytic [34]. For this reason, the analytic form of *x*(*t*) is first created by *y*(*t*) = *x*(*t*) +*jH*[*x*(*t*)], where *H*[·] denotes the Hilbert transform of a real signal.
2. Estimate the instantaneous frequency of *k* th harmonic, *f*(*t*), by searching the local maximum in the time-frequency representation of *x*(*t*).
3. Construct the kernel function *s*_0_(*t*) via numerical integration of the right-hand side of Equation (4), where *f*_0_ takes the average value of *f*(*t*) in this paper.
4. Apply the GFT to the analytic signal *y*(*t*), by doing so, *x_k_*(*t*) is mapped into a constant frequency signal *x̂_k_*(*t*). As *x̂_k_*(*t*) is well separated with other signal components in frequency domain, it can be extracted by conventional band-pass filtering.
5. Apply the inverse GFT to the filtered *x̂_k_*(*t*), and then extract the real part of *x̂_k_*(*t*). In this way, the waveform of *x_k_*(*t*) is finally obtained.

## 3. The Procedure of Tacholess Envelope Order Analysis Technique

**Figure 2.**
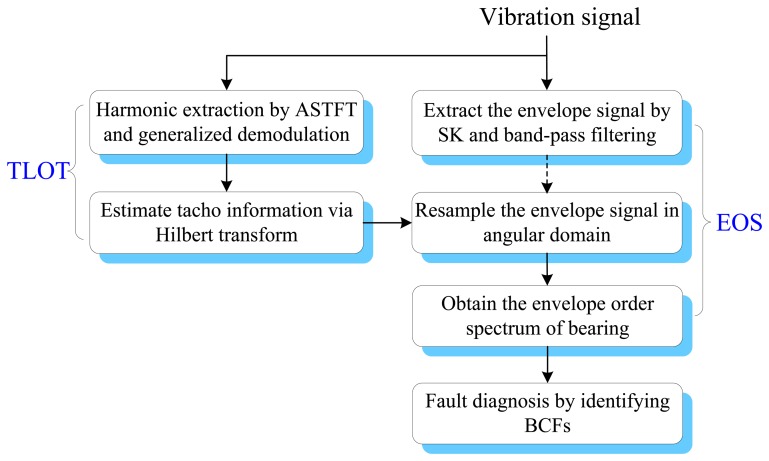
Flow chart of the proposed tacholess envelope order analysis technique.

To effectively diagnose the faults of rolling element bearings under variable operating conditions without a tachometer, a tacholess envelope order analysis (TLEOA) technique is proposed in this paper. The principle of TLEOA is mainly composed of two parts, *i.e.*, tacholess order tracking (TLOT) and envelope order spectrum (EOS). TLOT recovers the tacho information from the vibration signal in an adaptive way. EOS transforms the envelope to angular domain, which makes the BCFs more clear and discernible in the envelope order spectrum. The flow chart of the TLEOA technique is shown in Figure 2, and the implementation is discussed in detail as follows.

Step 1. Extract the envelope signal of impulses by using spectral kurtosis and band-pass filtering.
  Spectral kurtosis (SK) has been proven efficient in detecting incipient faults from large noise, which provides a means of determining which frequency bands contain a signal of maximum impulsivity [1,13]. As a result, SK is an effective tool to determine the centre frequency and bandwidth where the impulses hidden in. For the gear-bearing signals, the frequency components from gears have wide frequency spread and may contaminate the resonance frequency band of bearing. This interference will affect the performance of SK significantly. For this reason, it is usually advantageous to remove such discrete frequencies by order tracking and synchronous averaging before SK [1]. However, for rotor-bearing signals, the rotating frequency and its harmonics of the rotor are mainly located in the low frequency region, which is well separated with the resonance frequency band of bearing. In these applications, the SK could be directly applied to the raw signal to find out the optimal filtering band. Once the filtering band is obtained, a band-pass filter could be designed so as to recover the envelope signal of impulses from the broad-band background noise.
Step 2. Estimate the IF of *k* th harmonic by adaptive short-time Fourier transform.
  As discussed in Section 2.2, the accurate instantaneous frequency (IF) of *k*th harmonic is required before generalized demodulation. There are several algorithms available for IF estimation [35–38]. In this article, the adaptive short-time Fourier transform (ASTFT) based IF estimation method is employed [39]. The main advantage of this method is that the basis function of ASTFT can be adjusted to match the analyzed signal, hence a more concentrated time-frequency representation could be obtained. Moreover, this method is robust to measurement noise, thus it can produce reliable IF estimation results even under low SNR situation.
Step 3. Recover the instantaneous phase of the shaft via generalized demodulation and Hilbert transform.
Once the IF of *k*th harmonic is estimated, its waveform *x_k_*(*t*) can be extracted from overall signal by generalized demodulation as given in Section 2. Since the extracted *x_k_*(*t*) is a mono-component signal, its instantaneous phase *φ_k_*(t) can be calculated by the following equation:
  (5) $$\varphi_{k}(t)=\text{arc}\tan\left(\frac{H[x_{k}(t)]}{x_{k}(t)}\right)$$
  After that, the instantaneous phase of the shaft could be estimated according to Equation (1).
Step 4. Resample the envelope signal in angular domain.
  As discussed previously, the envelope signal of the impulses is non-stationary due to speed variation, which in turn leads to the smearing effect in the envelope spectrum. To deal with this problem, the envelope signal is resampled in angular domain according to the instantaneous phase information recovered from the vibration signal. In this way, the non-stationary envelope signal in time-domain can be transformed into a cyclo-stationary signal in the angular domain [40,41].
Step 5. Detect the bearing fault by identifying the BCFs in envelope order spectrum.
  Since the envelope signal has already been resampled (or order tracked), the traditional FFT-based methods can be effectively applied to that signal. By identifying of the BCFs in envelope order spectrum, the fault type of bearing can be determined.

## 4. Simulations

To validate the proposed method, a simulated signal is generated according to the vibration model for rolling element bearings [20]:

(6) $$x(t)=\sum_{i}A_{i}s(t-T_{i})+\sum_{n}B_{n}\cos\left(2\pi nf(t)+\beta_{n}\right)+n(t)$$

The simulated signal *x*(*t*) is composed of three terms. The first term represents a series of impulses excited by fault, where *A_i_* is the amplitude of the *i*th impulse and *T_i_* is the time of its occurrence. The second term represents the fundamental frequency and its harmonics of the shaft, which is caused by misalignment, eccentric or imbalance. In this term, *B_n_* and *β_n_* are the amplitude and initial phase of the *n*th harmonic, *f*(*t*) is the instantaneous rotating frequency of the shaft. The third term *n*(*t*) denotes the measurement noise.

In this simulation, we assume the outer race is keep fixed and the inner race is rotating with shaft. There is a local fault on the outer race, and the fault excited impulse is simulated by an exponentially decaying sinusoid as follows:

(7) $$s(t)=e^{-\alpha t}\sin(2\pi f_{r}t)$$

where *α* is the damping ratio of the impulse, which takes 500 Hz here; *f_r_* is the resonance frequency, which takes 2,000 Hz here. The ball pass frequency of outer race (BPFO) is assumed three times of the rotating frequency of shaft, then the average angular period of impulse is 360/3 = 120 degrees. Suppose there are three harmonics of shaft in the signal, and their amplitudes and phases are *B*_1_ = 0.3, *B*_2_ = 0.5, *B*_3_ = 0.4, *β*_1_ = π/6, *β*_2_ = −π/3, *β*_3_ = π/2, respectively. The bearing experiences a speed up and coast down process during the measurement, and the speed curve is given by Equation (8):

(8) f(t)=[250+400⋅sin(2π⋅0.125⋅t)]/60

**Figure 3.**
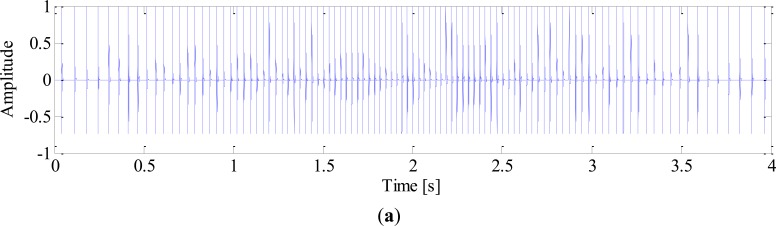

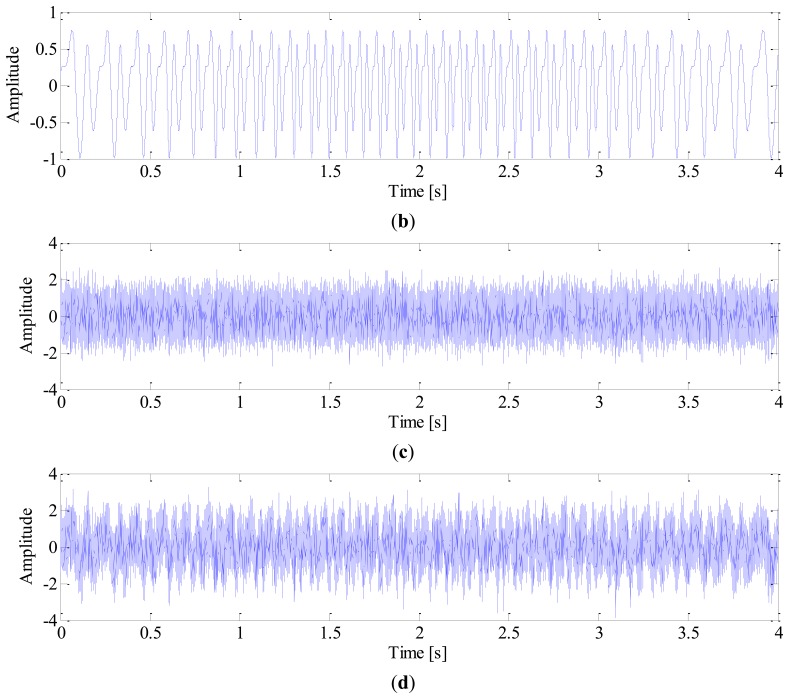
Simulated signal: (**a**) Impulse signal; (**b**) Harmonics of shaft; (**c**) Noise signal; (**d**) Mixed signal.

White noise is added to obtain a noisy signal with SNR of −3 dB. The sampling frequency is 10 k Hz and the time length of data is 4 s. The impulse signal, harmonics of shaft, noise signal and mixed signal are shown in Figure 3, respectively.

**Figure 4.**
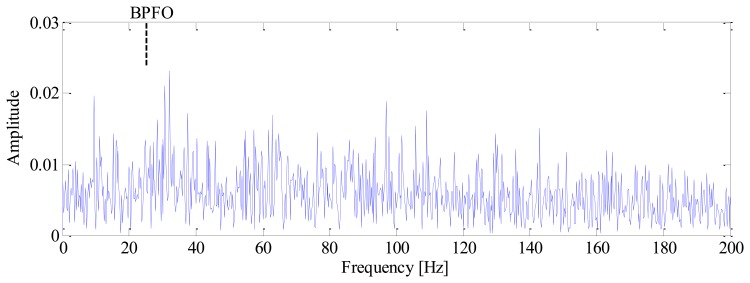
The conventional envelope spectrum of simulated signal.

The impulses generated by fault can hardly be observed in the mixed signal due to heavy noise. Moreover, the impulses are not equally spaced in time domain due to speed variation, which will bring difficulties to the conventional diagnostic methods based on constant speed. To demonstrate this problem, the conventional envelope analysis [1] is applied to the vibration signal, and the corresponding envelope spectrum is given in Figure 4. It is observed that the envelope spectrum is smeared when speed variation occurs, from which we cannot identify the BPFO. This drawback of conventional method decreases the accuracy and reliability of diagnostic results significantly.

For comparison, the proposed TLEOA technique is performed on the same simulated signal by the following steps.

**Figure 5.**
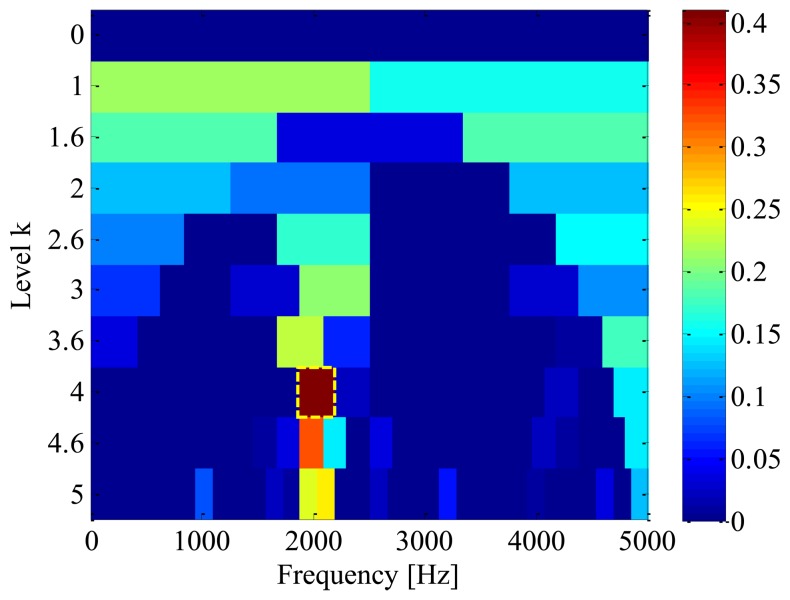
Kurtogram of simulated signal.

**Figure 6.**
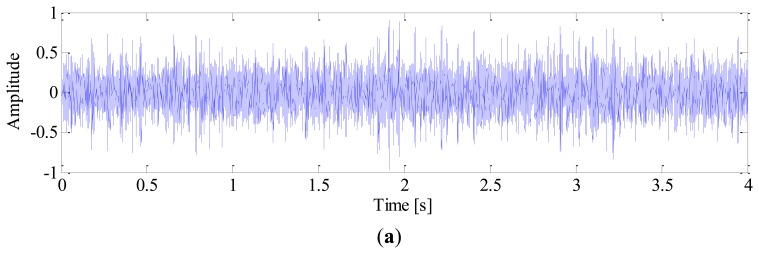

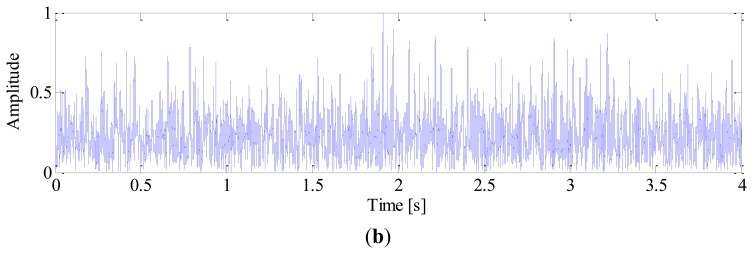
(**a**) Band-pass filtered signal; (**b**) The envelope signal of (a).

Firstly, in order to find out the optimal frequency-band for demodulation, a fast-kurtogram [8] based spectral kurtosis analysis is applied to the simulation signal. We chose to analyze the simulated signal with 5 decomposition levels, with a 1/3-binary tree. The corresponding kurtogram is displayed in Figure 5, from which the resonance frequency-band with centre frequency of 2031.25 Hz and bandwidth of 312.5 Hz can be identified clearly. With this information, an optimal band-pass filter is then designed to extract the impulses from the raw signal. Figure 6 illustrates the filtered signal and the corresponding envelope, respectively.

**Figure 7.**
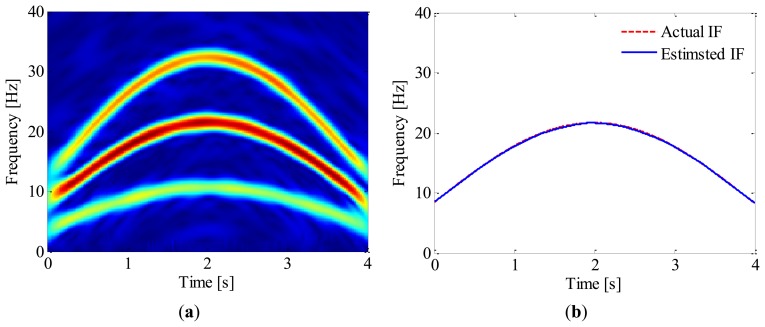
(**a**) ASTFT spectrogram zoomed in 0–40 Hz; (**b**) IF estimation result.

Subsequently, the time-frequency representation of the simulated signal is obtained by using ASTFT. Figure 7a illustrates the corresponding spectrogram zoomed in 0–40 Hz. From this figure, the fundamental and 2nd harmonic of bearing shaft can be identified clearly. Since the latter is energy-dominant, we decide to use its instantaneous phase to resample the envelope signal. The IF of 2nd harmonic is then estimated by searching the local maximum in the spectrogram. The IF estimation result is presented in Figure 7b; for comparison, the actual IF of 2nd harmonic is also plotted in the same figure. It is clear that the estimated IF agrees well with the actual one and the maximum estimation error is less than 1%.

**Figure 8.**
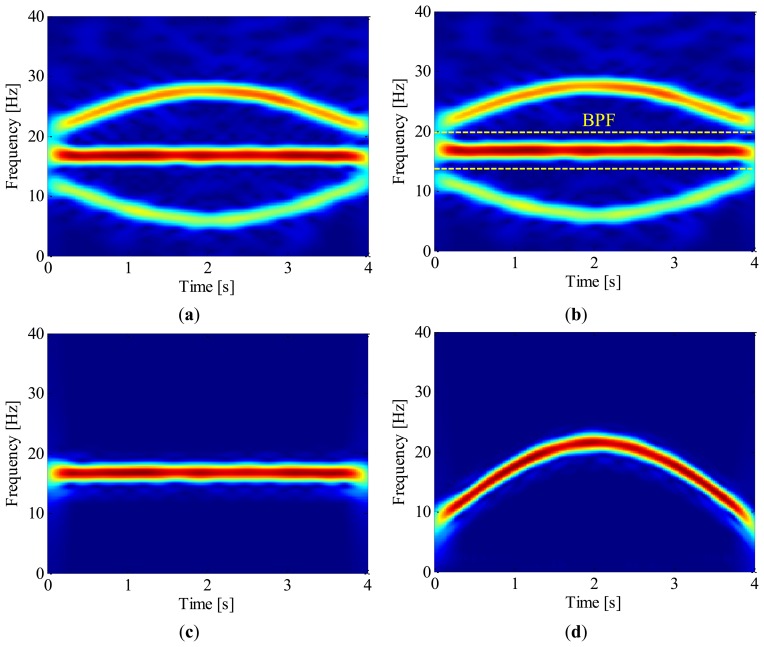
Extracting the 2nd harmonic by generalized demodulation: (**a**) Spectrogram of the generalized Fourier transformed signal; (**b**) Extracting the 2nd harmonic by BPF; (**c**) The 2nd harmonic is separated after BPF; (**d**) Restoring the 2nd harmonic by inverse GFT.

**Figure 9.**
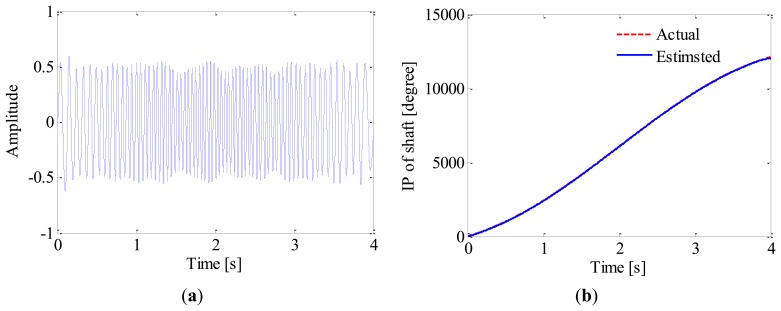
(**a**) Waveform of 2nd harmonic; (**b**) Instantaneous phase of shaft.

Once the IF of 2nd harmonic is estimated, generalized demodulation is further applied to extract its waveform from the raw signal. The exacting procedure is presented intuitively in Figure 8. Firstly, the kernel function of GFT, *s*_0_(*t*), is constructed by numerical integration. By multiplying the analytic signal with *s*_0_(*t*), the 2nd harmonic is mapped into a constant frequency signal in the transformed domain as shown in Figure 8a. A band-pass filter is then employed to extract the 2nd harmonic as illustrated in Figure 8b,c, and the half bandwidth takes 3 Hz so as to exclude the 1st harmonic. By inverse GFT, the waveform of 2nd harmonic is obtained as shown in Figure 9a. After that, the instantaneous phase of the shaft as presented in Figure 9b is obtained according to Equation (5) and Equation (1). It is reasonable to conclude that the proposed method could recover the instantaneous phase of the shaft accurately even without tachometer.

**Figure 10.**
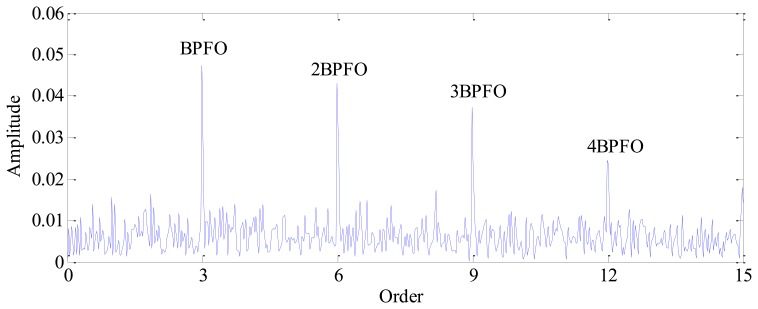
Envelope order spectrum obtained by proposed TLEOA technique.

Finally, the envelope signal is resampled according to the instantaneous phase recovered from the vibration signal. The spectral analysis is performed on this resampled signal and the corresponding envelope order spectrum is presented in Figure 10. Since the envelope signal has been transformed into angular domain, the smearing problem encountered in the conventional method, as illustrated in Figure 4, is eliminated effectively. It can be seen from Figure 10 that the BPFO = 3 orders (which means the BPFO is three times the rotating frequency of the bearing) and its harmonics are rather evident, which clearly indicates the existence of a fault on the outer race.

## 5. Application to Fault Detection of Locomotive Roller Bearings

In this section, the vibration signals collected from a locomotive bearing will be used to demonstrate the effectiveness of the proposed method.

**Figure 11.**
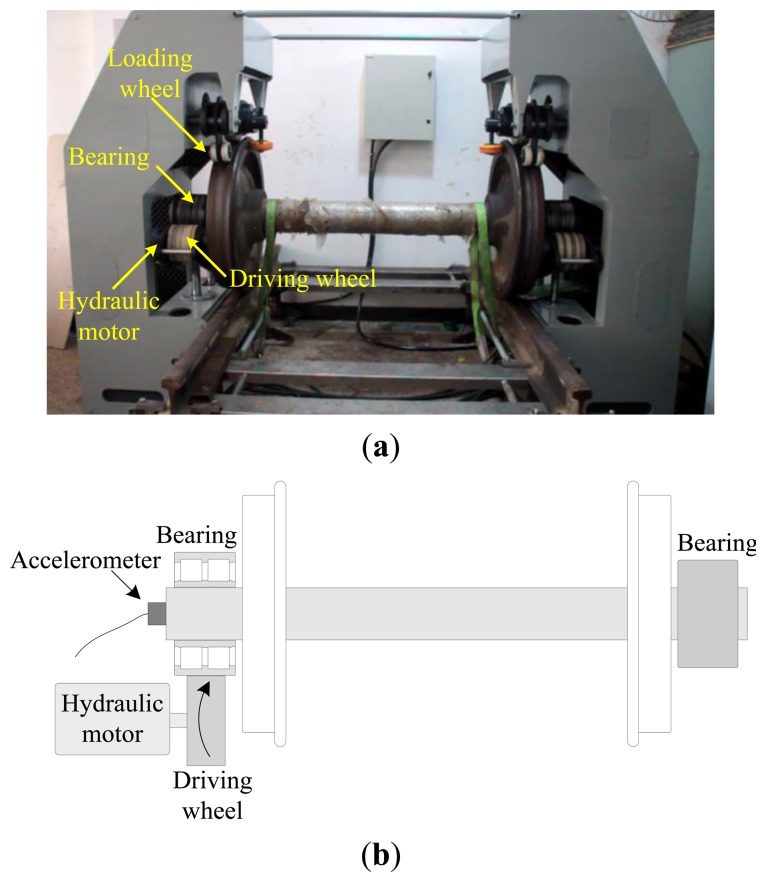
(**a**) The arrangement of the locomotive bearing test bench; (**b**) Schematic view.

The overview of the bearing test bench is given in Figure 11a. The outer race of the locomotive bearing is driven by a Nylon driving wheel which is connected to a hydraulic motor, while the inner race of the bearing is keep fixed in the measuring process. The nominal rotational speed of the hydraulic motor is 400 rpm. However, due to oil pressure fluctuation, the actual rotational speed can hardly keep constant. The geometric parameters of the bearing are listed in Table 1. According to those parameters, the bearing characteristic frequencies, including the ball pass frequency of inner race (BPFI), the ball pass frequency of outer race (BPFO) and the ball spin frequency (BSF), are calculated and given in Table 2. Since the bearing is not running with a constant speed, the normalized BCFs with respect to the rotating frequency of outer race in terms of order, are also listed in Table 2.

**Figure 12.**
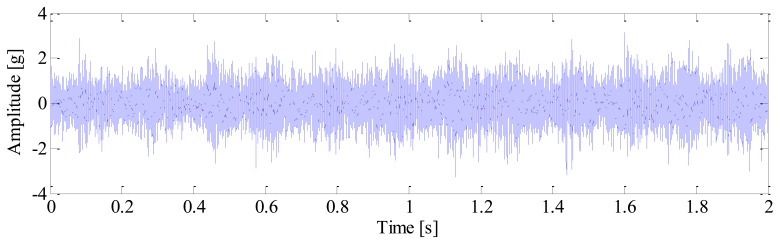
The raw vibration signal.

**Figure 13.**
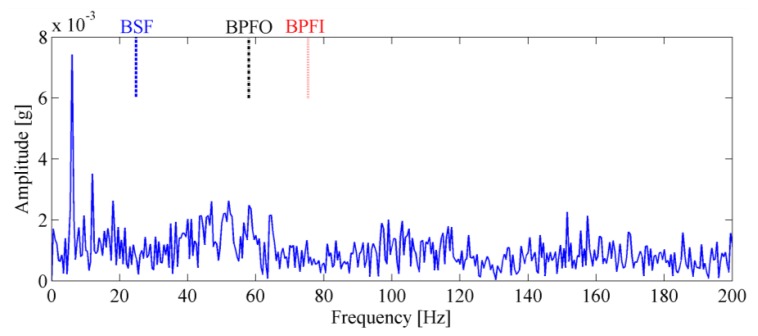
The envelope spectrum of the bearing signal.

A tri-axial PCB accelerometer with the model number 356A12 is mounted on the shaft end, as shown in Figure 11b, to collect the vibration signal generated by the locomotive roller bearing. Since the vertical vibrations are constrained by loading wheel and driving wheel of the test bench, their amplitudes are not as large as those of the horizontal vibrations. For this reason, the vibration signals of the horizontal direction are more sensitive to the damage and therefore they are analyzed in this paper. The sampling frequency is 76,800 Hz and the data length is 153,600. Figure 12 displays the collected vibration signal from a bearing with unknown fault. Due to heavy noise and interferences from other rotating components, we cannot see the impulses characterizing bearing faults from the raw signal. In order to assess the health status of the bearing, conventional envelope analysis is first applied to the vibration signal, and the corresponding envelope spectrum is illustrated in Figure 13. As discussed previously, the speed variation leads to smearing in the envelope spectrum, from which, none of the BCFs listed in Table 1 can be identified. As a consequence, the diagnostic result of conventional method is that the bearing is in healthy state. However, since some abnormal sound emitted from the bearing during the measurement, we suspect there may be some damages undetected. So we decide to reassess the condition of the bearing using the proposed TLEOA technique.

**Figure 14.**
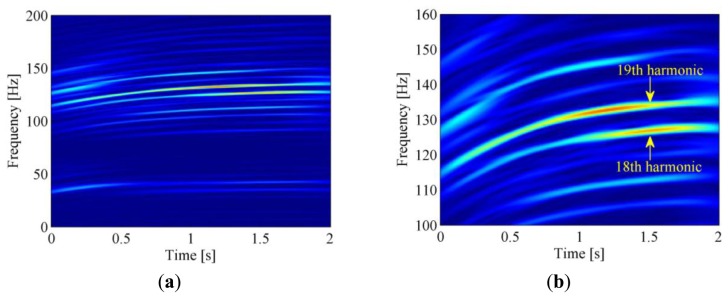
The ASTFT spectrogram of the bearing signal: (**a**) Overview; (**b**) Zoomed in 100–160 Hz.

Firstly, SK and band-pass filtering are performed on the raw signal to extract the envelope signal. Then the time-frequency representation of the raw vibration signal is obtained by ASTFT and the corresponding spectrogram is illustrated in Figure 14a. In this figure, two energy-dominant harmonics within 100–150 Hz can be seen clearly. By checking their center frequencies (133.9 Hz and 126.9 Hz at 1.5 s, respectively) and frequency interval (133.9 − 126.9 = 7 Hz) in the zoomed-in plot of Figure 14b, it is confirmed that they are the 18th and 19th harmonics of the driving wheel, respectively (126.9/7 ≈ 18, 133.9/7 ≈ 19). Since the 19th harmonic has greater energy, we try to use its instantaneous phase to resample the envelope signal.

**Figure 15.**
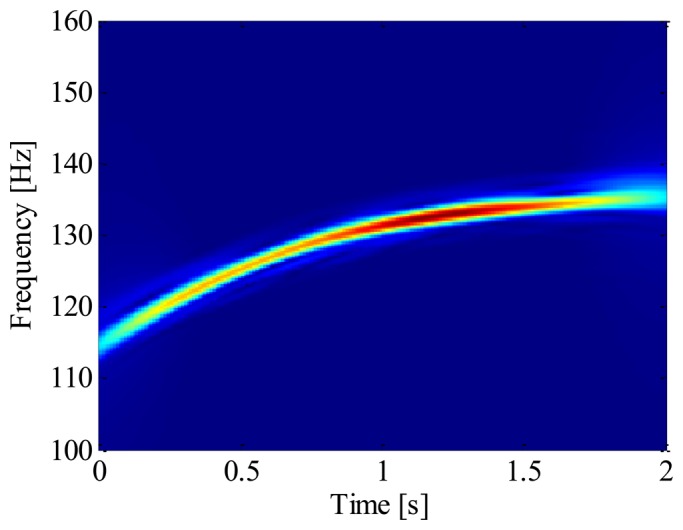
The ASTFT spectrogram of the extracted 19th harmonic.

The IF of 19th harmonic is estimated by ridge searching in the ASTFT spectrogram. After that, the 19th harmonic shown in Figure 15 is extracted from the raw signal by generalized demodulation method. Finally, the envelope signal is resampled according to the instantaneous phase of the extracted harmonic.

**Figure 16.**
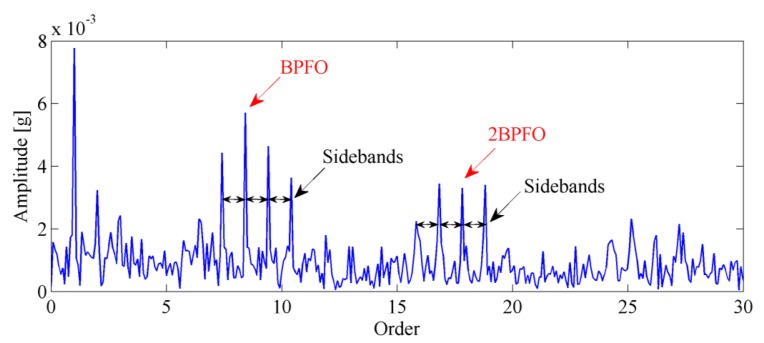
The envelope order spectrum obtained by proposed technique.

**Figure 17.**
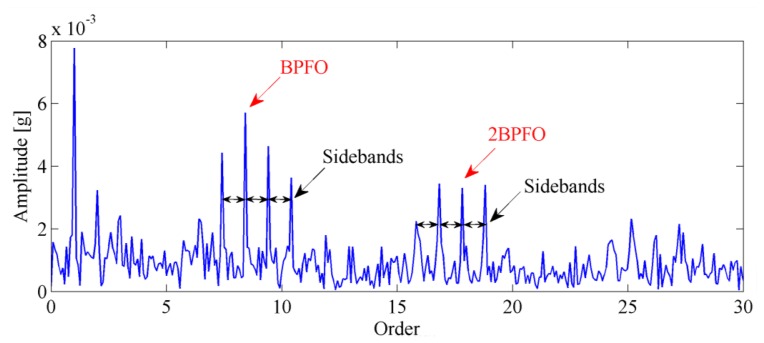
The spall fault on the outer race.

The envelope order spectrum obtained by the proposed method is illustrated in Figure 16. From this figure, the BPFO and its harmonics are clearly discriminated. Moreover, the sidebands caused by load modulation can also be detected. Those characteristics of envelope order spectrum clearly indicate the existence of a fault on the outer race of the bearing. Figure 17 shows the image of the disassembled bearing, from which, the spall fault in the outer race can be seen obviously.

**Figure 18.**
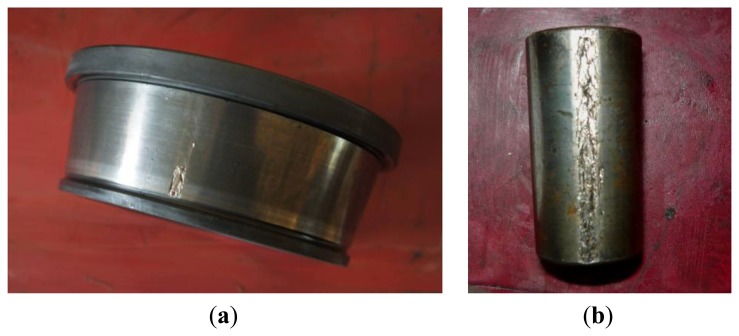
(**a**) Inner race fault; (**b**) Roller fault.

**Figure 19.**
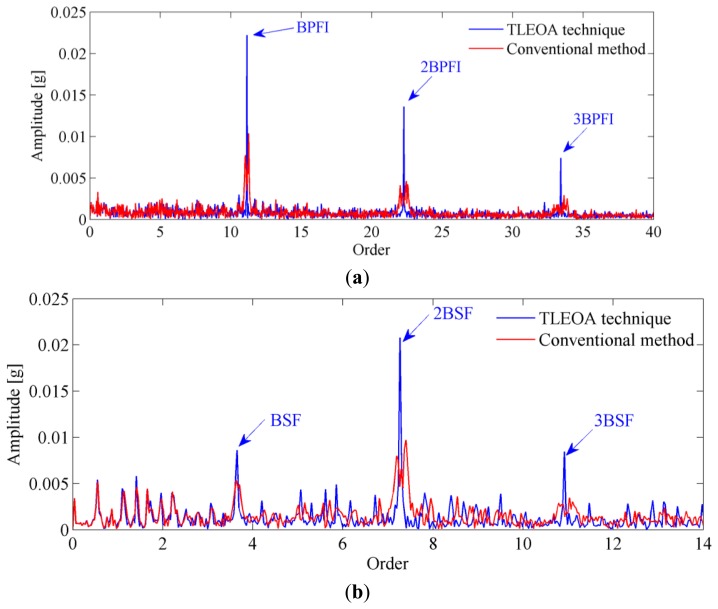
Comparison with conventional method: (**a**) Inner race fault detection; (**b**) Roller fault detection.

To further confirm the reliability of the proposed technique, the vibration signals from inner race and roller faults as shown in Figure 18 are also analyzed. The envelope order spectrums obtained by proposed technique are presented in Figure 19, respectively. For comparison, the envelope spectrums by conventional method are frequency normalized and plotted in the same figure. It can be seen from those figures that conventional method suffers from smearing problem when speed variation exists, which makes the amplitudes of BCFs decrease significantly. While the proposed TLEOA is free from smearing problem, and could recover the BCFs clearly and sharply even under speed variation cases. For a quantitative comparison, the enhancement in amplitudes of BCFs by the proposed technique is calculated and listed in Table 3. It can be concluded from Table 3 that the proposed TLEOA provides better capabilities of bearing detection than conventional method.

## 6. Conclusions

A technique has been proposed to extract the fault information of rolling element bearings in the variable speed case. Some conclusions are drawn in this work as follows:

Generalized modulation is an effective non-stationary signal analysis tool, which is capable of extracting one particular harmonic of bearing shaft rotating frequency from the raw signal. By using the instantaneous phase information of the extracted harmonic, order tracking can be performed on the envelope signal without tachometer. Envelope order spectrum could exploit the cyclic feature from non-stationary envelope signals, thus the smearing problem encountered in conventional methods could be addressed effectively. By combining those advantages, the proposed tacholess envelope order analysis technique could not only extend the conventional envelope analysis to speed varying case, but also be carried out without the use of additional sensors, such as tachometer or encoder. The effectiveness of the proposed method is demonstrated by simulation and experimental results. It is shown that this method gives a more reliable diagnostic result than conventional technique and therefore it is a promising method for bearing fault diagnosis.

## Figures and Tables

**Table 1.** Geometric parameters of the roller bearing.

**Pitch Diameter (mm)**	**Roller Diameter (mm)**	**Contact Angle (degree)**	**Number of Rollers**
180	23.775	9	20

**Table 2.** BCFs of the bearing.

**Items**	**BCFs in Hz**	**BCFs in Order**
BPFO	57.97	8.695
BPFI	75.37	11.305
BSF	24.81	3.721

**Table 3.** The enhancement in amplitude by proposed TLEOA technique.

**Items**	**1st Harmonic**	**2nd Harmonic**	**3rd Harmonic**
BPFI	113.7% (6.6 dB)	194.0% (9.4 dB)	154.0% (8.1 dB)
BSF	60.5% (4.1 dB)	113.8% (6.6 dB)	207.1% (9.7 dB)
